# Prevalence and correlates of healthy lifestyle behaviors among early cancer survivors

**DOI:** 10.1186/s12885-015-2019-x

**Published:** 2016-01-05

**Authors:** Iris M. Kanera, Catherine A. W. Bolman, Ilse Mesters, Roy A. Willems, Audrey A. J. M. Beaulen, Lilian Lechner

**Affiliations:** Faculty of Psychology and Educational Sciences, Open University of the Netherlands, P. O. Box 2960, 6401 DL Heerlen, The Netherlands; CAPHRI School for Public Health and Primary Care, Maastricht University, P.O. Box 616, 6200 MD Maastricht, Maastricht The Netherlands

**Keywords:** Cancer survivors, Health behaviors, Physical activity, Nutrition guidelines, Smoking, Alcohol drinking, Self-efficacy, Behavior mechanisms

## Abstract

**Background:**

Healthy lifestyle behaviors have been demonstrated to be beneficial for positive health outcomes and the quality of life in cancer survivors. However, adherence to recommendations is low. More insight is needed in factors that may explain engagement in lifestyle behaviors to develop effective cancer aftercare interventions. This study assessed different factors, namely socio-demographic, cancer-related, psychological, social cognitive factors (attitude, social support, self-efficacy) and intention, in relationship to five lifestyle behaviors (smoking, physical activity, alcohol, and fruit and vegetable consumption).

**Methods:**

Early survivors of various types of cancer were recruited from eighteen Dutch Hospitals (*n* = 255). Distal factors (socio-demographic, cancer related, psychological), proximal factors (social cognitive), intention and five lifestyle behaviors (smoking, physical activity, alcohol, fruit and vegetable consumption) were assessed through a self-reported questionnaire. Cross-sectional analyses (correlations and regression analyses) were conducted.

**Results:**

The lifestyle of a small group (11 %) of the cancer survivors was coherent with all five health recommendations, the majority (>80 %) adhered to two, three of four recommendations, and only few (<7 %) adhered to one or none recommendation. The highest prevalence in followed recommendations have been detected in physical activity (87.4 %), refrain from smoking (82 %), and alcohol consumption (75.4 %). There was low adherence to the fruit recommendation (54.8 %) and to the vegetable recommendation (27.4 %). Only weak associations were found between the different behaviors. Each separate lifestyle behavior was influenced by different patterns of correlates. Self-efficacy, attitude, and intention were the strongest correlates in all examined behaviors, although with various contributions, while socio-demographic, cancer-related and psychological factors provided a much smaller contribution.

**Conclusions:**

Outcomes of engagement in healthy lifestyle behaviors were more positive in this study compared to other research in cancer survivors; however, there is room for improvements in adherence to all five lifestyle behaviors. Especially fruit consumption was poor and vegetable consumption even worse. Our findings emphasized that all examined lifestyle behaviors need to be encouraged, with taken into account that each lifestyle behavior may be influenced by a specific set of mainly social cognitive factors or intention.

## Background

A healthy lifestyle is of major importance for cancer survivors, since it has been shown that adherence to an increasing number of health recommendations may lower the risk of lifestyle related chronic diseases and may lead to a higher health related quality of life [1–5]. Moreover, unhealthy behaviors may have a negative impact on quality of life and cause new health problems such as cancer recurrence, new primary tumors and other chronic diseases [2, 6–11]. Health recommendations for cancer survivors include the following: achieve and maintain a healthy body weight (body mass index (BMI) within the range of 18.5 to 25.0 kg/m2), engage in at least 30 min of moderately intense physical activity per day at five or more days weekly, eat five servings of fruit and vegetables daily, avoid or limit alcohol consumption to up to two servings per day for men and one serving per day for women, and refrain from smoking [12–14]. Previous research suggested that adherence to physical activity recommendations might be the most important lifestyle behavior associated with lower mortality and higher quality of life in cancer survivors [10, 11, 15].

Recent research showed that cancer survivors do not adhere consistently to these health recommendations. More than half is overweight, less than half adhere to physical activity recommendations, about only one fifths adhere to fruit en vegetable recommendations, about 90 % do not smoke, and approximately 90 % of cancer survivors adhere to the alcohol recommendations [1, 10, 16, 17]. Broadly, similar results were found in people without a history of cancer [18–21]. Until now, research about the adherence to a combination of health behaviors showed mixed results: European studies report about 10-28 % of the cancer survivors followed zero or one recommendation, about one third adhered to two, and also about one third adhered to three, and about 10-23 % adhered to four recommendations [3, 4]. American studies reported even lower adherence scores to multiple health behaviors [1, 21, 22]. In comparison, research conducted in the general population among older adults indicated that most of them followed three or more lifestyle recommendations (86 %) [23], suggesting less adherence among cancer survivors compared to the general population. Considering that cancer survivors are at increased risk of cancer recurrence and lifestyle-related chronic diseases, adhering to multiple lifestyle recommendations is however very important for the health related quality of life of this specific group. This underlines the need to understand which factors explain the different health behaviors and the adherence to an increasing number of lifestyle recommendations. Furthermore, possible correlations among lifestyle behaviors need to be identified to understand possible mutual influences.

As theoretical framework for our search into factors that relate to a healthy lifestyle among cancer survivors, we applied the central thoughts and concepts from social cognitive models: the Reasoned Action Approach, the Attitude-Social influence-Efficacy (ASE) model and its successor the Integrated Model for Behavior Change (I-Change-Model) [24–27]. These models assume that behavior can be predicted by a behavioral intention, which is influenced by proximal factors (social cognitive concepts: attitudes, perceived social influences and self-efficacy expectancies), which in turn can be influenced by more distal factors. In the current study, as distal factors we applied socio-demographic, psychological, and cancer related factors.

In recent years, studies identified correlates of physical activity, however, less is known about the correlates of the other lifestyle behaviors. Regarding physical activity, besides cancer related variables (fatigue, physical side effects), attitude, self-efficacy, social support and intention were important correlates in explaining physical activity in cancer survivors [28, 29]. Additionally, exercise history could be identified as important predictor of exercise adherence. However, for intention, perceived behavior control, age, gender, education, physical fitness and psychological features the findings were inconsistent [30, 31]. Considerably fewer publications described possible correlates of healthy diet, alcohol consumption, and smoking in cancer survivors. Madlensky et al. (2008) identified motivation and self-efficacy as strong predictors of the dietary pattern in breast cancer survivors [32]. Current smoking in cancer survivors was correlated with younger age, lower education and income, and greater alcohol consumption, while quitting after cancer diagnosis was associated with having a smoking related type of cancer [33].

The aims of the present study were 1) to assess the prevalence of lifestyle behaviors and the adherence to recommendations in early cancer survivors, 2) to examine correlations between the different health behaviors and 3) to explore the contribution of socio-demographic, cancer-related, psychological features, social cognitive factors and intention to explain lifestyle behaviors and adherence to recommendations. To our knowledge, this is the first study, exploring the combined contribution of distal factors (enclosing cancer specific socio-demographic and psychological factors), more proximal factors (such as attitude, social support, self-efficacy), and intention, derived from social cognitive models to explain five lifestyle behaviors and adherence to recommendations in early cancer survivors with various types of cancer.

## Methods

We conducted a cross-sectional survey among early cancer survivors with various types of cancer. This study was approved by the Ethics Review Board on Research (cETO) of the Open University of the Netherlands, Heerlen, The Netherlands. The study was carried out in accordance with the American Psychological Association’s Ethics Code and the Declaration of Helsinki, 2013 [34]. No further approval by the Medical Research Ethics Committee (MREC) was necessary, because present study did not fall under the Medical Research Involving Human Subjects Act (WMO).

### Study population

Cancer survivors from Dutch outpatient departments of internal medicine, oncology, and urology were invited to participate. Required sample size of the most extensive multiple regression analysis was *N* ≥ 160. Inclusion criteria were: adults (>18 years) diagnosed with and treated for one type of cancer with no sign of recurrence at the last control visit; surgery, chemotherapy and/or radiation therapy as primary treatment, which has been completed at least 6 weeks and up to one year ago. Cancer survivors with severe medical, psychiatric of cognitive problems that would interfere with participation were excluded from the study.

### Study procedure

Eighteen hospitals in the South of the Netherlands were approached for recruitment of participants. Medical staff of eight hospitals agreed and recruited cancer survivors in the period from November 2012 until January 2013. Two recruitment strategies were used: 1) selection of cancer survivors through record review by (research) nurses or 2) personal invitations during outpatient clinic visits with oncologist, urologist, or nurse practitioner. Potentially eligible participants received an information letter, an informed consent form, and a survey booklet. A reminder letter followed 2 weeks later. Cancer survivors, who agreed to participate, were asked to provide written informed consent, to complete the questionnaires and to return these documents to the researchers in an enclosed pre-paid envelope.

### Measurements

All measurements concerned self-report questionnaires.

#### Lifestyle outcome measures

Physical activity was assessed using the International Physical Activity Questionnaire Short Form (IPAQ Short) [35–37]; standardized questions from Dutch Measuring Instruments for Research on Smoking and Smoking Cessation were used to measure smoking behavior [38]; Nine items from the Dutch standard questionnaire on nutrition measurements were used to determine vegetable and fruit consumption [39, 40]; alcohol consumption was assessed by using four items from the Dutch standard questionnaire on alcohol consumption [39]. Table 1 provides an overview of these measurements and their properties.

**Table 1:** Lifestyle outcome measurements

Behavior	Questionnaire/example question	Categories/scales	Items	Item-range	Score-range
Physical Activity^a^	IPAQ Short last 7 days self-administered format	Walking	2		MET-min/week
		Moderate intensive activity	2		
		Vigorous intensive activity	2		
Smoking	“Do you currently smoke?”	Current smoking behavior	1	0-1	0-1
	“Did you smoke in the past?”	History of smoking (quit smoking before/ after cancer diagnosis)	1	0-1	0-1
Alcohol consumption	Dutch standard questionnaire on alcohol consumption	Number of days and glasses of alcohol on weekdays and weekends	4	0-6	0-4
		Binge drinking^b^	1	1-8	0-7
Vegetable and fruit consumption^c^	Dutch standard questionnaire on nutrition	Number of servings fruit/vegetable (spoons, pieces, glasses) per day and number of days per week	9	1-9	0-7

*Note*: IPAQ Short: International Physical Activity Questionnaire Short Form; MET: Metabolic Equivalent of Task

^a^ ≥ 600 MET-min/week corresponds to ≥ five days per week performing any combination of walking, moderate or vigorous physical activities

^b^ ≥ Six servings of alcohol during one day

^c^ Vegetable consumption was expressed in grams per day. The total score for fruit consumption was the number of servings of fruit per day (up to 100 g fruit may be replaced by fruit juice)

#### Socio-demographic measures

Socio-demographic items were measured using standard questions on age, gender, marital status, education level (‘low’: lower vocational education, medium general secondary education; ‘medium’: secondary vocational education, higher general secondary education; ‘high’: higher vocational education, university education), income level (‘below average’: < €1800 per month; ‘average’: > €1800 and < €2200 per month; ‘above average’: > €2200 per month), employment status (‘working’: self-employed, in paid employment; ‘not working’: unemployed, retired, unable to work).

#### Cancer-related measures

Standard questions were used to assess cancer-related factors. Type of cancer was subsequently categorized into breast, colon, and other types; because of insufficient numbers of the separate types of cancer for appropriate statistical analyses (see footnote Table 3). Type of treatment was categorized into surgery alone, surgery & chemotherapy, surgery & radiation, surgery, chemotherapy & radiation, and other types for the same reason. Aftercare participation was dichotomized (yes/no). Information on length and weight were used to calculate the body mass index (BMI).

#### Psychological measures

Table 2 provides an overview of the psychological measures and their properties. Quality of life (QoL) was assessed by using the European Organisation for Research and Treatment of Cancer (EORTC QLQ-C 30) [41–43]. Anxiety and depression were measured by applying the Hospital Anxiety and Depression Scale (HADS) [44–46]. Adjustment to cancer was assessed using the Mental Adjustment to Cancer Scale (MAC) [47–49]. Illness perception was assessed with the Brief Illness Perception Questionnaire (Brief IPQ) [50, 51]. The items of the latter questionnaire were adjusted to focus on recovery from cancer, and item 4 (treatment control) was deleted to achieve an acceptable internal consistency (increase Cronbach’s alpha from .61 to .75 after removing item 4). Problem solving orientation was measured by using the Short Social Problem Solving Inventory-Revised (SPSI–R:S) [52].

**Table 2:** Psychological outcome measures

Concept	Instrument	Subscales used	Items	Score-range	α	Higher scores indicates
Quality of life	EORTC	Global health status	2	0-100	.88	Better overall health and quality of life
	QLQ-C30					
		Physical functioning	5	0-100	.72	Better functioning
		Role functioning	2	0-100	.86	Better functioning
		Emotional functioning	4	0-100	.86	Better functioning
		Cognitive functioning	2	0-100	.70	Better functioning
		Social functioning	2	0-100	.70	Better functioning
		Fatigue	3	0-100	.87	Higher level of problems
		Nausea and vomiting	2	0-100	.52	Higher level of problems
		Pain	2	0-100	.82	Higher level of problems
		Dyspnea	1	0-100		Higher level of problems
		Insomnia	1	0-100		Higher level of problems
		Appetite loss	1	0-100		Higher level of problems
		Constipation	1	0-100		Higher level of problems
		Diarrhea	1	0-100		Higher level of problems
		Financial difficulties	1	0-100		Higher level of problems
						Higher level of problems
Anxiety, depression	HADS	Anxiety	7	0-21	.84	More morbidity
		Depression	7	0-21	.80	More morbidity
Adjustment to cancer	MAC	Positive adjustment			.78	More positive adjustment
		Fighting spirit	16	16-64		
		Avoidance	1	1-4		
		Negative adjustment			.84	More negative adjustment
		Helplessness/Hopelessness	6	6-24		
		Anxious preoccupation	9	9-36		
		Fatalism	8	8-32		
Illness perception	Brief IPQ	Consequences, Timeline, Personal control, Identity, Concern, Coherence, Emotional representation	7	0-70	.80	More threatening view of the illness
Problem solving orientation	SPSI–R:S	Positive problem orientation	5	0-4	.72	Positive outcome and self-efficacy expectancies, less emotional distress
		Negative problem orientation	5	0-4	.86	Negative outcome and self-efficacy expectancies, more emotional distress

*Note:* QLQ-C30: Quality of Life Questionnaire; HADS: Hospital Anxiety and Depression Scale; MAC: Mental Adjustment to Cancer Scale; Brief IPQ: Brief Illness Perception Questionnaire; SPSI–R:S: Short Social Problem Solving Inventory-Revised; α: Cronbach’s α

#### Social cognitive measures

Attitude, social support, self-efficacy, and intention for each lifestyle behavior were measured by using single items for the separate concepts consisting of 5-point scales with a score ranging from 1 to 5. Attitude was assessed with questions such as “Is it important for you to follow the nutrition guidelines?” Answer options were yes, very important (5), yes, important (4), not important/not unimportant (3), no, not important (2), no, not at all important (1). Social support was measured by asking questions such as “To what extent do you get support from people who are important to you, to exercise sufficiently?” Response options were always (5), often (4), sometime (3), seldom (2), never (1). Self-efficacy was assessed by asking questions such as “Is it easy or difficult for you to exercise according to the guidelines?” Answering choices were very easy (5), easy (4), not difficult/not easy (3), difficult (2) very difficult (1). Intention was measured by asking questions such as “Do you intend to eat 2 servings of fruit a day in the next 6 months?” Response options were yes, certainly (5), yes, probably (4), maybe/maybe not (3), no, probably not (2), no, certainly not (1). Prior research also applied similar items to measure social cognitive concepts [53–57].

### Statistical analyses

Analyses were conducted using SPSS 21. We used descriptive statistics to describe participant characteristics and the prevalence of health behaviors. For describing the adherence to separate recommendations, we constructed two categories (yes, no) for all five health behaviors.

Missing values were handled according to the questionnaire manuals. For the EORTC QLQ-C30, HADS, and MAC the permitted number of missing values was one. For the SHORT SPSI-R two missing values were permitted. The missing values were supplemented by using mean substitution, as recommended. Cases with missing values on days and time (physical activity), days and number of servings (nutrition and alcohol) were removed from analysis. For other measures, less than 5 % of the values were missing per value in a random pattern. We applied mean substitution for continuous covariates and for categorical covariates, we substituted the values of the modus.

To assess the contribution of the distal and proximal factors in explaining alcohol, vegetable, and fruit consumption, and physical activity we conducted four sequential multiple linear regression analyses [58]. The variables were entered in four entry steps based on the social cognitive models (e.g. Reasoned Action Approach [27], I-Change-Model [26]), the theoretical framework of the present study. The models prescribe an ordering of steps. This implies that socio-demographic and cancer-related factors were entered in order to control for their possible influence. Then, the psychological factors were entered in step 2 to evaluate what they add to the explanation of variance over and above the first set, the background variables. Subsequently, in step 3, the influence of attitude, social support, and self-efficacy were assessed above the two prior sets. Intention was added it in the last step, according to the assumptions of the social cognitive theories, that intention is influenced by the prior added proximal factors.

To explore the correlates of smoking behavior (smoking vs quitting) among former smokers and current smokers, we conducted sequential logistic regression analysis [58]. Never-smokers were excluded from this analysis. In the logistic regression analysis, we applied the same entry steps as described above. Results from sequential logistic regression analysis (*N* = 139) revealed large confidence intervals, due to the relative small number of participants and a large number of independent variables. Consequently, we conducted a second sequential logistic regression analysis, including fewer variables. The insignificant socio-demographic variables were removed, but core variables were entered in step 1 (age, gender, education level, type of cancer, and type of treatment). Significant psychological variables were added in entry step 2, such as the significant concepts from the EORTC QLQ-C30 (global health/QoL, cognitive functioning, social functioning, nausea /vomiting, insomnia, financial difficulties), and the subscales anxiety and depression from the HADS). In entry step 3 attitude, social support, and self-efficacy were added, and intention was added in the last step.

Furthermore, we were interested in the correlates to explain the overall degree of adherence to lifestyle recommendations. Therefore, we conducted sequential multiple regression analysis and applied the same protocol as described for the multiple regression analyses.

Moreover, correlations between the continuously measured lifestyle behaviors (alcohol, vegetable, fruit consumption, physical activity) were assessed, using Spearman’s correlation due to non-normally distributed data. Additionally, by conducting Chi-square tests among the five adherence scores we assessed the correlations between adherence to different health behaviors.

## Results

### Recruitment and characteristics of the sample

**Fig. 1:**
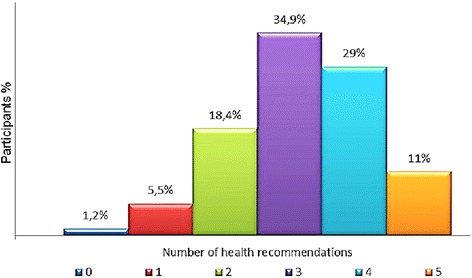
Adherence to lifestyle recommendations (*N* = 255). *Note:* The five recommendations relate to physical activity, not smoking, alcohol, fruit and vegetable consumption

In total, 455 cancer survivors were invited to participate in the study, 172 (37.8 %) cancer survivors declined participation, 22 (4.8 %) cancer survivors did not meet the inclusion criteria, and six (1.3 %) respondents did not return the informed consent form. We included 255 (56 %) respondents in the analysis. Participants’ descriptive characteristics are displayed in Table 3. The prevalence of lifestyle behaviors is displayed in Table 4, and the adherence to recommendations is shown in Fig. 1.

**Table 3:** Characteristics of the sample (*N* = 255)

Variable		Variable	
Age years (SD)	60.6 (10.7)	Type of cancer	
Gender		Breast, n (%)	150 (58.8)
Female, n (%)	193 (70.7)	Colon, n (%)	51 (20)
Marital status		Other, n (%)^a^	54 (21.1)
Living with partner, n (%)	217 (86.5)	Type of treatment	
Educational level		Surgery alone, n (%)	32 (12.6)
Low, n (%)	137 (54.6)	Surgery and chemotherapy, n (%)	55 (21.7)
Medium, n (%)	47 (18.7)	Surgery and radiotherapy, n (%)	46 (18.1)
High, n (%)	67 (26.3)	Surgery, chemo- & radiotherapy, n (%)	92 (36.2)
Employment status		Other, n (%)	29 (11.4)
Not working, n (%)	158 (64)	Participation in aftercare	
Income level		Yes, n (%)	134 (53)
Below average, n (%)	51 (21.1)	Number of weeks after treatment, mean (SD)	26.5 (12.7)
Average, n (%)	70 (28.9)	HADS, mean, (SD)	8.2 (6.7)
Above average, n (%)	121 (50)	HADS anxiety, mean (SD)	4.7 (3.9)
BMI, mean (SD)	26.7 (9.4)	HADS depression, mean (SD)	3.5 (3.5)
< 18,5: underweight, n (%)	1 (0.4)	MAC	
18,5-25: healthy weight, n (%)	113 (45.7)	Positive adjustment, mean (SD)	51.1 (7.0)
25-30: overweight, n (%)	95 (38.5)	Negative adjustment, mean (SD)	29.6 (7.0)
30-35: obesity, n (%)	25 (10.1)	IPQR, mean (SD)	32.5 (10.9)
> 35: extreme obesity, n (%)	13 (5.3)	SPSIR	
EORTC QLQ-C30		Positive problem orientation, mean (SD)	2.4 (0.8)
Global health status, mean (SD)	78.1 (16.5)	Negative problem orientation, mean (SD)	1.1 (0.9)
Physical functioning, mean (SD)	85 (15.3)	Alcohol Attitude, mean (SD)	2.2 (1.3)
Role functioning, mean (SD)	79.4 (23.8)	Social support, mean (SD)	2.2 (1.5)
Emotional functioning, mean (SD)	80.1 (20.4)	Self-efficacy, mean (SD)	3.6 (1.3)
Cognitive functioning, mean (SD)	80.6 (22)	Intention, mean (SD)	2.4 (1.5)
Social functioning, mean (SD)	82.8 (21.4)	Physical Activity Attitude, mean (SD)	4.6 (0.5)
Body Image, mean (SD)	82.3 (22.8)	Social support, mean (SD)	3.6 (1.2)
Fatigue, mean (SD)	27 (23.9)	Self-efficacy, mean (SD)	3.5 (1.1)
Nausea and Vomiting, mean (SD)	3.3 (10.3)	Intention, mean (SD)	4.7 (0.7)
Pain, mean (SD)	15.9 (22.6)	Nutrition Attitude, mean (SD)	4.1 (0.7)
Dyspnea, mean (SD)	12 (21.9)	Social support, mean (SD)	3.1 (1.3)
Insomnia, mean (SD)	26.1 (28)	Self-efficacy, mean (SD)	3 (0.9)
Appetite loss, mean (SD)	6.2 (16.6)	Intention vegetable consumption, mean (SD)	4.2 (1.0)
Constipation, mean (SD)	8.2 (18.4)	Intention fruit consumption, mean (SD)	4.0 (1.1)
Diarrhea, mean (SD)	7.5 (20)		
Financial difficulties, mean (SD)	10.6 (22.5)		

*Notes:* n: numbers of participants; SD: standard deviation; BMI: Body Mass Index; EORTC: European Organisation for Research and Treatment of Cancer; QoL: Quality of Life; HADS: Hospital Anxiety and Depression Scale;MAC: Mental Adjustment to Cancer scale; IPQ: Illness Perception Questionnaire; SPSIR-R:S Short Social Problem Solving Inventory-Revised

^a^ other types of cancer were prostate (9 %); Non-Hodgkins’s lymphpma (5.9 %), ovarian (3.1 %); bladder (1.2 %); cervix (0.4 %); Hodgkins’s lymphpma (0.4 %)

**Table 4:** Lifestyle behaviors of the sample

Behavior			Meet recommendations	
	Mean (SD)	Median (IQR)	Yes, n (%)	No, n (%)
Smoking (*n* = 250)				
Never			108 (43.2)	
Former			97 (38.8)	
Current				45 (18)
Alcohol consumption (*n* = 244)^1^			184 (75.4)	60 (24.6)
Never			58 (22.8 %)	
Social (*n* = 186)			126 (67.7 %)	
Excessive (*n* = 186)				60 (32.3 %)
Male drinkers (*n* = 60)^1.1^			39 (65 %)	21 (35 %)
Female drinkers (*n* = 126)^1.2^			87 (69 %)	39 (31 %)
Vegetable consumption^2^ (*n* = 248)	167.7 (90.8)	150 (107.2 -203.6)	68 (27.4)^2.1^	180 (72.6)
Fruit consumption^3^ *(n* = 252)	1.8 (1.1)	2 (1-2)	138 (54.8)^3.1^	114 (45.2)
Physical activity in MET-min/week^4^				
Walking (*n* = 234)	1299.3 (1188.5)	924 (396 – 2079)		
Moderate *(n* = 232)	1600.6 (1623.8)	1200 (210 – 2400)		
Vigorous (*n* = 235)	962.9 (1734.5)	0 (0 -1440)		
Total MET-min/week (*n* = 247)^4.1^	3657.6 (3293.4)	2613 (1284 – 5145)	216 (87.4)^4.2^	31 (12.6)

Notes: *n*: numbers of participants; SD: standard deviation; IQR: interquartile range; MET: Metabolic Equivalent of Task

^1^ number of alcohol consumptions per week; ^1.1^ male: ≤ 14 drinks per week; ^1.2^ female: ≤ 7 drinks per week

^2^ vegetable consumption per day in grams; ^2.1^ ≥ 200 g vegetables per week

^3^ number of fruit servings (à 100 g) a day. Up to 100 g fruit may be replaced by 150 g of fruit juice. ^3.1^ at least 2 servings of fruit per week

^4^ MET-min/week = metabolic equivalent*minutes per week; ^4.1^ Total MET-min/week = walking + moderate + vigorous; ^4.2^ > 600 MET min p/week

### Correlations between the different lifestyle behaviors

We explored mutual correlations between the continuously measured lifestyle behaviors (alcohol, fruit, vegetable consumption, physical activity). Fruit consumption was significantly positively correlated to vegetable consumption, *r*_s_ = .24, *p* < .001, and we found a negative relationship between fruit consumption and alcohol consumption *r*_s_ = -.14, *p* < .05, which indicated that as fruit consumption was higher, alcohol consumption was lower. No other significant correlations were found.

Furthermore, we explored correlations between adherence (yes, no) to the five different health recommendations and found a statistically significant association between adherence to the smoking and fruit consumption recommendations (*χ*^2^ (1) = 6.285, *p* < .05), however, the effect size represented a low association (*Cramer’s V* = .16, *p* < .05). Crosstabs showed, that in smokers, 37.8 % met the fruit recommendations, while in non-smokers (former smoker or never-smoker), 58.3 % adhered to the fruit recommendations. No further associations were found between other adherence scores.

### Correlates of lifestyle behaviors and adherence to recommendations

The results of the regression analyses to explain lifestyle behaviors and adherence to recommendations are presented in Table 5 en Table 6.

**Table 5:** Correlates of lifestyle behaviors

	Lifestyle behavior														
	Number of alcohol consumption			Number of vegetable consumption			Number of fruit consumption			Amount of physical activity			Nonsmoking		
	(*N* = 223)			(*N* = 225)			(*N* = 228)			(*N* = 225)			(*N* = 141)^a^		
Variable	B	(95 % CI)	*p*	B	(95 % CI)	*p*	B	(95 % CI)	*p*	B	(95 % CI)	*p*	ExpB	(95 % CI)	*P* ^*c*^
Age	. 037	(-.15; .23)	.698	−1.307	(-2.70; .09)	.067	.007	(-.01; .02)	.367	−13.723	(-25.94; -1.51)	.028*	.936	(.86; 1.02)	.127
Female gender	−5.805	(-11.13; -4.77)	.033*	7.579	(-32.98; 48.14)	.713	.211	(-.20; .63)	.315	−11.993	(-361.07; 337.08)	.946	.394	(.04; 4.45)	.399
Marital status															
Without partner	ref			ref			ref			ref					
With partner	−4.986	(-10.75; .73)	.089	24.032	(-17.65; 65.72)	.257	.174	(-.25; .60)	.421	−122.543	(-478.92; 233.83)	.498			
Education															
Low	ref			ref			ref			ref			ref		.198
Medium	−1.165	(-5.60; 3.72)	.605	14.521	(-17.78; 64.82)	.376	-.019	(-.35; .31)	.907	−176.621	(-451.64; 98.40)	.207	2.664	(.52; 13.75)	.242
High	−2.370	(-6.64; 1.90)	.274	18.004	(-1.71; 49.72)	.264	.075	(-.25; .40)	.644	−215.435	(-474.80; 43.93)	.103	5.451	(.72; 41.44)	.101
Income															
Above average	ref			ref			ref			ref					
Average	−2.646	(-6.42; 1.13)	.168	−19.041	(-47.66; 9.58)	.191	-.118	(-.41; .17)	.423	71.279	(-169.63; 312.19)	.560			
Below average	−4.183	(-9.78; 1.41)	.142	−6.040	(-47.44; 35.36)	.774	.022	(-.40; .45)	.917	−77.931	(-431.78; 275.19)	.654			
Cancer type															
Other	ref			ref			ref			ref			ref		.096
Breast	−1.244	(-7.97; 5.48)	.716	−1.055	(-51.79; 49.68)	.967	-.046	(-.56; .47)	.862	137.614	(-291.14; 566.37)	.527	.489	(.02; 12.11)	.662
Colon	−1.859	(-8.18; 4.46)	.562	5.333	(-42.80; 53.47)	.827	.002	(-.49; .49)	.995	15.656	(-390.56; 421.88)	.939	.045	(.00; 1.04)	.053
Treatment															
All^b^	ref			ref			ref			ref			ref		.554
Surgery alone	1.914	(-4.18; 8.01)	.536	−6.261	(-51.72; 39.20)	.786	-.077	(-.54; .39)	.745	−67.186	(-443.93; 309.56)	.725	.483	(.04; 5.73)	.565
Surgery, chemo	.991	(-3.40; 5.38)	.657	-.259	(-33.72; 33.20)	.988	−0.52	(-.39; .29)	.764	56.566	(-223.74; 3236.87)	.691	3.975	(.38; 41.21)	.247
Surgery, radiation	.228	(-4.88; 5.34)	.930	−15.436	(-52.36; 21.49)	.411	.031	(-.35; .21)	.872	−43.457	(-358.83; 271.92)	.786	.471	(.06; 3.68)	.473
Other	-.041	(-7.96; 7.88)	.992	-.947	(-60.33; 58.44)	.975	-.144	(-.74; .45)	.633	−70.914	(-559.88; 418.05)	.775	1.275	(.04; 43.16)	.893
Aftercare															
No	ref			ref			ref			ref					
Yes	1.914	(-5.00; 2.39)	.487	−15.766	(-43.27; 11.74)	.260	-.070	(-.35; .21)	.622	−60.426	(-291.29; 170.44)	.606			
Time after treatment	.991	(-.236; .026)	.117	1.053	(.09; 2.02)	.032*	.003	(-.01; .01)	.500	.787	(-7.26; 8.83)	.874			
BMI	-.257	(-.654; .14)	.203	1.654	(-1.32; 4.63)	.274	-.013	(-.04; .02)	.376	−7.844	(-33.21; 17.53)	.453			
Glob. Health/ QoL	.044	(-.10; .12)	.543	-.412	(-1.48; .67)	.451	-.002	(-.01; .01)	.732	.246	(-8.74; 9.23)	.957	.926	(.86; 1.00)	.052
Physical funct.	.008	(-.17; .18)	.929	-.676	(-1.99; .46)	.313	-.007	(-.02; .01)	.314	5.129	(-6.24; 16.50)	.374			
Role funct.	–.076	(-.20; .05)	.199	.521	(-.38; 1.42)	.254	.006	(-.00; .02)	.164	4.379	(–3.05; 11.81)	.246			
Emotional funct.	–.078	(–.20: .05)	.226	.263	(–.71; 1.24)	.593	.003	(–.01; .01)	.554	7.327	(–.94; 15.59)	.082			
Cognitive funct.	–.013	(–.12; .09)	.796	–.079	(–.86; .70)	.840	–.002	(–.01; .01)	.600	.192	(–7.84; 6.51)	.953	.957	(.92; 1.00)	.055
Social funct.	.070	(–.05; .19)	.234	–.466	(–1.32; .39)	.285	–.003	(–.01; .01)	.488	–.661	(–7.84; 6.51)	.856	1.046	(1.00; 1.09)	.038*
Body Image	.016	(–.07; .10)	.703	.010	(–.65; .66)	.977	.000	(–.01; .01)	.935	1.977	(–3.43; 7.38)	.471			
Fatigue	.046	(–.07; .16)	.436	.269	(–.63; 1.16)	.554	.001	(–.01; .01)	.744	7.732	(.30; 12.15)	.041*			
Nausea,vomiting	–.043	(–.23; .15)	.651	–.071	(–1.56; 1.42)	.925	.000	(–.02; .01)	.957	−2.743	(–14.78; 9.29)	.654	.945	(.89; 1.01)	.081
Pain	–.034	(–.13; .06)	.472	.169	(–.56; .90)	.650	.001	(–.01; .01)	.784	6.229	(.31; 15.16)	.039*			
Dyspnea	–.025	(–.11; .06)	.553	–.428	(–1.07;.21)	.188	–.004	(–.01; .00)	.277	.010	(–5.19; 5.21)	.997			
Insomnia	–.062	(–.13; .00)	.058	.214	(–.27; .70)	.382	.000	(–.00; .01)	.866	.850	(–3.13; 4.83)	.674	.977	(.95; 1.01)	.108
Appetite loss	.033	(–.08; .15)	.576	–.168	(–1.05; .72)	.708	.001	(–.01; .01)	.805	4.505	(–2.91; 11.91)	.223			
Constipation	–.036	(–.12; .03)	.452	–.374	(–1.10; .35)	.310	–.005	(–.01; .00)	.186	−5.298	(–11.12; .53)	.074			
Diarrhea	.030	(–.13; .06)	.503	.111	(–.59; .81)	.754	.003	(–.01; .01)	.383	.151	(–5.57; 5.87)	.959			
Financial probl.	.001	(–.09; .09)	.987	–.339	(–.99; .31)	.302	–.001	(–.01; .01)	.827	.024	(–5.44; 5.49)	.993	.977	(.95; 1.01)	.157
Anxiety	–.080	(–.78; .62)	.820	–.084	(–5.43; 5.26)	.975	–.008	(–.06; .05)	.760	41.203	(–3.24; 85.65)	.069	.682	(.50; .93)	.015*
Depression	.170	(–.60; .94)	.662	–.204	(–6.03; 5.62)	.945	–.027	(–.09; .03)	.365	−33.248	(–80.72; 14.22)	.169	1.187	(.89; 1,58)	.236
Pos. adjustment	–.057	(–.32; .20)	.667	2.297	(.34; 4.26)	.022*	.013	(–.02; .03)	.198	12.872	(–3.001; 28.75)	.111			
Neg. adjustment	–.149	(–.48; .18)	.373	−1.992	(–4.40; .42)	.105	.005	(–.02; .03)	.714	6.782	(–13.48; 27.04)	.510			
Illness perception	–.034	(–.24; .17)	.738	.782	(–.71; 2.28)	.303	.010	(–.01; .03)	.189	−4.296	(–16.86; 8.27)	.501			
PPO	1.324	(–1.05; 3.70)	.273	−4.790	(–22.60; 13.02)	.596	–.001	(–.18; .18)	.991	−78.106	(–225.46; 69.24)	.297			
NPO	.457	(–1.75; 2.66)	.684	2.240	(–14.26; 18.74)	.789	–.078	(–.25; .09)	.360	−82.287	(–222.29; 57.73)	.248			
Attitude	1.522	(–.32; 3.36)	.105	17.076	(–1.98; 36.13)	.079	–.016	(–.21; .18)	.872	192.876	(–19.74; 405.49)	.075	5.707	(1.83; 17.7)	.003**
Social support	–.004	(–.01; .00)	.290	–.012	(–.19; .17)	.894	0.00	(–.00; .00)	.710	–.111	(–1.62; 1.40)	.884			
Self–efficacy	−1.532	(–2.81; –.25)	.019*	11.342	(–3.70; 26.36)	.138	.070	(–.09; .23)	.378	181.637	(54.71; 308.56)	.005*	2.583	(1.42; 4.69)	.002**
Intention	.479	(–1.02; 1.98)	.530	36.980	(23.16; 50.81)	.000**	.597	(.48; .72)	.000**	137.551	(–23.15; 298.25)	.093	.583	(.58; 1.56)	.852
Constant B (SE)	35.146 (20.40)			−105.53 (151.51)			−1.63 (1.54)			−2456.07 (1356.25)			2.96 (5.07)		
R^2^	.297			.415			.514			.312					
Sig. F Change	.530			.000			.000			.093					
Cox & Snell R^2^													.518		
Nagelkerke R^2^													.725		
Model *X* ^2^													102.87		
*p*													.000		

*Note:* From Sequential Multiple Regression (continuous outcomes) and Sequential Logistic Regression (smoking) entry step 4 is displayed. Forced entry (enter) method was used; *Abbreviations*: ExpB: odds ratio; ref: reference group; BMI: Body Mass Index; EORTC: European Organisation for Research and Treatment of Cancer; QoL: Quality of Life; HADS: Hospital Anxiety and Depression Scale; neg./pos. adjustment from MAC: Mental Adjustment to Cancer Scale; IPQ: Illness Perception Questionnaire; SPSIR–R:S Short Social Problem Solving Inventory–Revised; Chemo: chemotherapy; PPO: positive problem orientation; NPO: negative problem orientation

^a^Dependent variable encoding: if participant is former smoker 1; if participant is current smoker 0; never–smokers were excluded

^b^all = surgery + chemotherapy + radiation

^c^ *p*–value of Wald test is presented

*p < 0.05; **p < 0.01

**Table 6:** Correlates of adherence to recommendations (*N* = 236)

	Adherence to an increasing number of lifestyle recommendations											
	Model 1			Model 2			Model 3			Model 4		
Variable	B	(95 % CI)	*p*	B	(95 % CI)	*p*	B	(95 % CI)	*p*	B	(95 % CI)	*p*
Age	.000	(-.02; .02)	.962	.004	(-.13; .02)	.610	-.006	(-.02; .01)	.441	-.010	(-.02; .01)	.201
Female gender	.655	(.18; 1.13)	.007	.686	(.18; 1.19)	.008	.398	(-.60; .86)	.088	.462	(.02; .90)	.039*
Marital status: with partner	.566	(.08;1.06)	.024	.460	(-.55; .98)	.080	.284	(-.17; .74)	.221	.391	(-.44; .83)	.078
Education, low = ref												
Medium	.200	(-.19; .59)	.307	.215	(-.19; .62)	.291	.035	(-.33; .40)	.850	-.019	(-.37; .33)	.915
High	.601	(.24; .96)	.001	.534	(.15; .92)	.006	.029	(-.34; .40)	.877	.117	(-.33; .47)	.517
Income, above average = ref.												
Average	.067	(-.28; .42)	.703	-.021	(-.38; .33)	.907	-.063	(-.38; .25)	.695	-.030	(-.33; .27)	.844
Below average	.062	(-.40; .52)	.789	.037	(-.48; .56)	.889	-.203	(-.66; .26)	.384	-.083	(-.52; .36)	.709
Cancer type, other = ref												
Breast	.459	(-.12; 1.04)	.122	.281	(-.35; .91)	.381	.130	(-.43; .69)	.646	.046	(-.47; .60)	.813
Colon	.291	(-.26; .84)	.296	.224	(-.37; 82)	.461	.087	(-.41;.62)	.744	.145	(-.36; .65)	.570
Treatment; all^1^ = ref												
Surgery alone	.076	(-.47; .60)	.805	-.004	(-.57; .56)	.988	.128	(-.39; .64)	.626	.033	(-.46; .52)	.894
Surgery + chemo	.049	(-.31; .50)	.644	.053	(-.37; .47)	.803	.025	(-.35; .40)	.897	.027	(-.33; .39)	.882
Surgery + radiation	-.467	(-.90; -.04)	.034	-.459	(-.91; -.01)	.046	-.209	(-.69; .11)	.157	-.264	(-.66; .14)	.195
Other	.577	(-.11; 1.26)	.100	.573	(-.48; 1.44)	.123	.123	(-.53; 78)	.711	.107	(-.52; .73)	.736
Participating in aftercare	-.083	(-.41; .24)	.611	.006	(-.33; .35)	.971	-.123	(-.42; .18)	.420	-.105	(-.39; .18)	.472
Time after treatment	.009	(-.00; .02)	.098	.010	(-.01; .02)	.109	.009	(-.00; .02)	.091	.012	(.00; .02)	.024*
BMI	.021	(-.01; .06)	.230	.017	(-.02; .05)	.347	.013	(-.02; .05)	.445	.006	(-.03; .04)	.734
Glob. Health/QoL	-	–	–	–.012	(–.03; .01)	.080	–.010	(–.02; .00)	.086	–.009	(–.02; .00)	.115
Physical funct.	–	–	–	–.003	(–.02; .02)	.740	–.004	(–.02; .01)	.604	–.004	(–.02; .01)	.619
Role funct.	–	–	–	.014	(.03; .03)	.015	.010	(.01;.02)	.036	.010	(.00; .02)	.027*
Emotional funct.	–	–	–	.001	(–.01; .01)	.854	.006	(–.01; .02)	.320	.007	(–.00; .02)	.184
Cognitive funct.	–	–	–	–.012	(–.02; –.00)	.008	–.009	(–.02; –.00)	.030	–.009	(–.02; –.00)	.026*
Social funct.	–	–	–	–.004	(–.01; .01)	.493	–.004	(–.01; .01)	.364	–.004	(–.01; .01)	.400
Body Image	–	–	–	.002	(–.01; .01)	.658	.002	(–.01; .01)	.657	.002	(–.01; .01)	.554
Fatigue	–	–	–	.001	(–.01; .01)	.922	.000	(–.01; .01)	.980	.001	(–.01; .01)	.779
Nausea en Vomiting	–	–	–	–.010	(–.03; .01)	.254	–.010	(–.03; .01)	.198	–.005	(–.02; .01)	.517
Pain	–	–	–	.002	(–.01; .01)	.713	.005	(–.00; .01)	.243	.006	(–.00; .01)	.140
Dyspnea	–	–	–	–.006	(–.01; .00)	.124	.002	(–.01; .01)	.620	.001	(–.01; .01)	.799
Insomnia	–	–	–	–.002	(–.01; .00)	.576	.001	(–.01; .01)	.998	.001	(–.01; .01)	.992
Appetite loss	–	–	–	–.002	(–.01; .01)	.759	–.001	(–.01; .01)	.856	–.001	(–.01; .01)	.869
Constipation	–	–	–	–.006	(–.01; .00)	.202	–.002	(–.01; .01)	.653	–.002	(–.01; .00)	.544
Diarrhea	–	–	–	–.001	(–.01; .01)	.811	.001	(–.01; .01)	.862	–.001	(–.01; .01)	.824
Financial difficulties	–	–	–	–.005	(–.01; .00)	.268	–.003	(–.01; .01)	.983	–.002	(–.01; .01)	.531
Anxiety	–	–	–	–.033	(–.10; .03)	.325	–.007	(–.07; .05)	.818	–.018	(–.07; .04)	.531
Depression	–	–	–	–.016	(–.09; .05)	.652	–.049	(–.11; .01)	.127	–.024	(–.09; .04)	.445
Positive adjustment	–	–	–	.023	(.00; .05)	.048	.025	(.01; .05)	.016	.020	(.00; .04)	.045*
Negative adjustment	–	–	–	.007	(–.02; .05)	.648	.026	(–01; .05)	.058	.023	(–.00; .05)	.095
Illness perception	–	–	–	.005	(–.01; .02)	.573	.003	(–.01; .02)	.693	.002	(–.01; .02)	.825
PPO	–	–	–	.042	(–.16; .24)	.684	–.003	(–.19; .018)	.975	.006	(–.17; .18)	.944
NPO	–	–	–	.088	(–.12; .29)	.394	.115	(–.07; .30)	.216	.089	(–.09; .26)	.317
Alcohol: Attitude	–	–	–	–	–	–	.084	(–.03; .20)	.158	.020	(–.13; .17)	.783
Social support	–	–	–	–	–	–	.000	(.00; .00)	.184	.000	(.00; .001)	.266
Self–efficacy	–	–	–	–	–	–	.052	(–.06; .16)	.344	.053	(–.05; .16)	.306
Nutrition: Attitude	–	–	–	–	–	–	.373	(.17; .58)	.000	.265	(.06; .47)	.010*
Social support	–	–	–	–	–	–	–.001	(–.00; .00)	.301	–.001	(–.00; .00)	.295
Self–efficacy	–	–	–	–	–	–	.112	(–.06; .28)	.200	–.037	(–.21; .14)	.671
Physical Activity: Attitude	–	–	–	–	–	–	–.184	(–.46; .09)	.193	–.157	(–.44; .12)	.269
Social support	–	–	–	–	–	–	–.001	(–.00; .00)	.170	–.001	(–.00; .00)	.206
Self–efficacy	–	–	–	–	–	–	.104	(–.07; .27)	.227	.080	(–.09; .25)	.347
Smoking: Attitude							.153	(–.07; .38)	.184	.100	(–.13; .34)	.387
Social support	–	–	–	–	–	–	.020	(–.06; .10)	.623	.000	(–.08; .08)	.993
Self–efficacy	–	–	–	–	–	–	.323	(.17; .47)	.000	.330	(.19; 48)	.000**
Intention												
Alcohol cons.	–	–	–	–	–	–	–	–	–	.066	(–.05; .18)	.373
Vegetable cons.	–	–	–	–	–	–	–	–	–	.112	(–.04; .26)	.144
Fruit cons.	–	–	–	–	–	–	–	–	–	.263	(.13; .39)	.000**
Physical activity	–	–	–	–	–	–	–	–	–	.045	(–.16; .25)	.668
Smoking	–	–	–	–	–	–	–	–	–	–.009	(–.12; .10)	.874
Constant B (SE)	.932 (.77)			.795 (1.85)			−2.728 (1.78)			−3.72 (1.72)		
R^2^	.172			.289			.502			.567		
Sig. F Change	.000			.109			.000			.000		

*Note:* From Sequential Multiple Regression (continuous outcomes) and Sequential Logistic Regression (smoking) entry step 4 is displayed. Forced entry (enter) method was used. *Abbreviations*: ExpB: odds ratio; ref: reference group; BMI: Body Mass Index; EORTC: European Organisation for Research and Treatment of Cancer; QoL: Quality of Life; HADS: Hospital Anxiety and Depression Scale; MAC: Mental Adjustment to Cancer Scale; IPQ: Illness Perception Questionnaire; SPSIR–R:S Short Social Problem Solving Inventory–Revised; Chemo: chemotherapy; PPO: positive problem orientation; NPO: negative problem orientation; R^2^:correlation coefficient squared

^1^all = surgery + chemotherapy + radiation

^2^Dependent variable encoding: if participant is former smoker 1; if participant is current smoker 0 *p*–value of Wald test is presented

*p < 0.05; **p < 0.01

#### Alcohol consumption

Being male (*p* = .033) and lower self-efficacy toward adherence to the alcohol recommendation (*p* = .019) were correlated to a higher alcohol consumption. Less problems of insomnia (*p* = .058) contributed to a lesser extent to a higher alcohol consumption. Before intention was added to the model, higher levels of attitude and lower self-efficacy contributed significantly.

#### Vegetable consumption

Significant correlates of a higher vegetable consumption were: 1) a stronger intention toward adhering to the vegetable recommendation (*p* = .000), 2) higher scores on positive mental adjustment (*p* = .022), 3) a longer period since completion of primary treatment (*p* = .032), and, to a smaller extent, lower age (*p* = .067). A higher attitude and self-efficacy were significantly correlated with vegetable consumption before intention was added to the model.

#### Fruit consumption

A stronger intention toward adherence to the fruit recommendation was the only significant correlate in explaining a higher fruit consumption (p = .000). Before intention was added to the model, lower levels of depressive symptoms, and higher self-efficacy contributed significantly.

#### Physical activity

Significant correlates in explaining a higher amount of physical activity were 1) younger ages (*p* = .028), 2) higher scores on self-efficacy toward adherence to the physical activity recommendation (*p* = .005), 2) more pain (*p* = .039), more fatigue (*p* = .041). Before intention was added to the model, higher levels of attitude and self-efficacy also contributed significantly.

### Not smoking

1) A more positive attitude toward not smoking (*p* = .003), 2) higher self-efficacy toward not smoking (*p* = .002), 3) lower levels of anxiety (*p* = .015), and 4) better social functioning (*p* = .038) were significantly correlated to not smoking among (former) smokers. Lower scores on global health/QoL (*p* = .052), lower scores on cognitive functioning (*p* = 0.55), and not having colon cancer (*p* = .053) contributed to a smaller extent.

#### Adherence to lifestyle recommendations

Significant correlates in explaining adherence to an increasing number of lifestyle recommendations were 1) a more positive intention toward following fruit (*p* = .000) recommendation, 2) higher scores on self-efficacy toward not smoking (*p* = .000), a more positive attitude toward following the nutrition recommendations (*p* = .010), and 3) three psychological factors (role functioning, *p* = .027; cognitive functioning, *p* = .026; positive mental adjustment to cancer, *p* = .045). In addition, a longer period after completing primary cancer treatment (*p* = .024) and female gender (*p* = .39) contributed to the adherence to lifestyle recommendations.

## Discussion

This cross-sectional study assessed the prevalence and correlates of five lifestyle behaviors in early cancer survivors. Additionally, contributing factors to explain the extent of adherence to lifestyle recommendations were assessed, from which only little evidence is available up to date. The special feature of this study is, that for the first time both, distal and proximal factors, derived from social cognitive theories, were assessed. In all analyses, the required number of participants, in terms of power, has been achieved. Valuable information was gained about important factors that may explain engagement in lifestyle behaviors and the extent of adherence to recommendations. The highest prevalence in followed recommendations have been detected in physical activity (87.4 %), refrain from smoking (82 %), and alcohol consumption (75.4 %). Low prevalence was found in adherence to the fruit recommendation (54.8 %) and, in particular in adherence to the vegetable recommendation (27.4 %).

### Physical activity

The proportion of participants meeting the physical activity recommendations (87.4 %) were much higher than results earlier reported [1, 16, 59]. In these studies, however, a different measurement instrument was used, which might explain the discrepancy. Our results are rather consistent with results from studies, which also used the IPAQ Short form; however, over-reporting might have been occurred [35, 60, 61]. An additional explanation for the fairly high level of physical activity might be the relatively good health of the participants. The sample characteristics (Table 1) showed rather high scores on the functioning scales as well as low scores on the symptom scales of the EORTC QLQ-C30, and low scores on the HADS. In addition, more than half of the sample used some form of cancer aftercare, which often has a strong emphasis on physical activity. From the individuals who were engaged in aftercare, almost 50 % were supported by an oncology physiotherapist or participated in a rehabilitation program including physical exercises. This might also partly explain the high level of PA among our sample of survivors.

Higher scores on self-efficacy lower ages, and, more pain, and more fatigue were the only significant correlates of a higher level of physical activity. Causal directions cannot be determined, but a possible explanation for the positive relationships between pain respectively fatigue and a higher level of physical activity could be, that pain and fatigue might have been reasons to get supervised by an (oncological) physiotherapist, or to follow a rehabilitation program. In the Netherlands, guidelines to cope with pain and fatigue are characterized by an active approach (gradually building up physical activity).

As described before, physical activity is an important modifiable lifestyle behavior, which can have an impact on health outcomes in cancer survivors. Even though most of the cancer survivors meet the recommendations in our study, in clinical practice, attention should be given to the maintenance and if possible, to a gradual increase of physical activity.

### Smoking

Of our sample, 18 % were current smoker, which is a higher rate of smokers compared to findings from other research [33, 62, 63]. Most of the former smokers quitted before cancer diagnosis, and half of the current smokers intended to quit within six months. The strongest correlates of not smoking were a higher self-efficacy, a more positive attitude toward nonsmoking, lower anxiety and better social functioning, while in other research, where social cognitive and psychological variables were not considered, younger age, lower education/ income, greater alcohol consumption, and cancer type were correlated with current smoking [33]. However, qualitative results of Berg et al. [64], confirmed that a positive attitude towards quitting may help to (remain) quit, and that feelings of anxiety and low self-efficacy were reasons to continue smoking, which corresponds to our results. Additionally, addiction and habit were also mentioned as important reasons to continue smoking. However, our study did not confirm their result, that depressive symptoms were correlated with continued smoking, possibly due to the low prevalence of depressive symptoms in our sample. Besides above mentioned findings, concepts of addiction and habit and a possible interaction with other risk behaviors (e.g. alcohol consumption) should be taken into consideration in further research. Because of the increased health risk of continued smoking, the high rate of motivated current smokers, and limited research in this field, further exploration of predictors and the development of programs to (remain) quit smoking for cancer survivors are needed.

### Alcohol consumption

Among alcohol drinkers, more than one third drank more than recommended, and 18.7 % preformed binge drinking (six or more servings a day, 1-3 times per month or even more frequently), which is considerably more than reported in other studies [62, 65, 66]. Possibly, people might not be aware of their excessive alcohol consumption and its long-term risk [9, 67, 68]. Earlier studies in older adults reported that alcohol consumption was related to positive sensations among older adults [69, 70]. Our finding, that low self-efficacy was associated with higher alcohol consumption might possibly be explained by the difficulty of breaking a particular drinking habit, assuming that a substantial number of participants consumed more than recommended, and thus drank regularly, and as discussed above, alcohol consumption might be accompanied by positive short term consequences. Given the long-term health risks, an increase of awareness and knowledge about personal (excessive) alcohol consumption and its consequences should be pursued in cancer survivors. It should be considered that our sample included never-drinkers, social drinkers and excessive drinkers, who possibly could be regarded as distinct groups.

### Vegetable and fruit consumption

Vegetable and fruit consumption were low in our sample, however, consistent or higher than in American cancer survivors [1, 16, 71]. Compared to European cancer survivors, especially vegetable consumption was considerably lower [65, 72, 73]. These low prevalence rates clearly demonstrate that the vegetable and fruit consumption can be greatly improved.

In nutrition recommendations and studies, vegetable and fruit consumption often are treated and presented as one single behavior, although there are differences in the prevalence and consumption of fruit and vegetables, e.g. in the Netherlands, vegetables are mostly a part of the main meals and fruit is often eaten as a snack between meals or as a desert. Our study showed only a small correlation between vegetable and fruit consumption and the factors associated with both behaviors were different, which advocates for treating vegetable and fruit consumption as two different types of behavior. A longer period after completing primary cancer treatment was correlated with a higher amount of vegetable consumption, but not with fruit consumption. The preparation of vegetables could take some effort, and possibly, cancer survivors might spend more effort in the preparation of meals including vegetables, the more time passed after the cancer treatment with possible side effects. Furthermore, the sense of taste could be affected during the cancer treatment and improve again afterwards. Possibly, this also could be a reason for a temporary change in diet. However, evidence is limited yet about correlates and predictors of vegetable and fruit consumption in cancer survivors.

In the present study, the strongest correlates in vegetable and fruit consumption were positive intentions, while being women and having a higher education were found to be correlated to meeting vegetable and fruit recommendation in other research [21]. Furthermore, we identified that more excessive alcohol drinkers and smokers were less likely to adhere to the fruit recommendation. The latter might be explained by assuming that smokers possibly smoke at times when nonsmokers eat fruit (e.g. during break times at work). These results confirm prior findings that risk behaviors among adults tend to cluster [74]. Moreover, it is shown that combinations or clustering of risk behaviors might be involved with additional health risks [75].

To disentangle separate determinants of vegetable and fruit consumption, more specific research is needed. In clinical practice, attention should be given to vegetable and fruit consumption to increase the intake in cancer survivors, preferably tailored to personal attitudes, self-efficiency expectations, and intentions.

### Adherence to recommendations

In our study, the adherence to recommendations (Fig. 1) was overall more positive in comparison with other studies [1, 3, 22]. Higher scores on attitude, self-efficacy, and intention of some of the lifestyle behaviors were the strongest correlates with adherence to an increasing number of recommendations (Table 6). The strong association between self-efficacy toward nonsmoking and adherence to recommendations could be explained by the presence of never-smokers (43.2 %) in our sample.

Not much is known about contributing factors in explaining adherence to an increasing number of lifestyle recommendations in cancer survivors, yet. We found that positive mental adjustment contributed (*p* = .045), what could be in line with findings from other research, reporting that emotional benefit-finding related to cancer was positively associated with engagement in several health behaviors [76]. Although the two concepts are not the same, we could assume that cancer survivors who are able to cope more positively with their situation might be more likely to be involved in healthier lifestyle behaviors. However, a direction en causality of this association cannot be determined in this study. We emphasize again, that especially for cancer survivors it may be important to live as healthy as possible. Therefore, more insight is needed in the determinants of engagement in as much as possible healthy lifestyle behaviors, and, furthermore, cancer aftercare programs should aim to target multiple lifestyle behaviors.

### Different patterns of correlates

For each separate lifestyle behavior we found different prevalence and different patterns of correlates. In accordance with the assumptions of social cognitive theories, we identified proximal variables and intention as strongest correlates in all examined behaviors, although with variations in contribution. Our results confirm theoretical assumptions [27], that the relative contribution of attitudes, self-efficacy and social influences can differ from one person to another and from one behavior to another. Regarding the distal factors, we found notably less, but also different patterns of correlations between the lifestyle behaviors. Overall, subscales of the EORTC QLQ-C30 provided the most influential distal factors, although the contribution of all distal factors (socio-demographic, cancer-related, psychological) was considerably lower than the contribution of the proximal factors and intention. It would be interesting to investigate a possible predicting role of the distal factors and possible mediation effects of the proximal factors in longitudinal research.

### Limitations

This study was subject to some limitations. Due to the cross-sectional design, no causal relationships and directions of associations could be determined. Furthermore, the collected data were based on self-report questionnaires. In particular, self-reported outcomes of lifestyle behaviors should be interpreted carefully. In addition, the results of his study might not be generalizable to all cancer survivors, because more than half of the sample has been women with breast cancer. Even though, cancer type and gender had limited correlates in explaining the lifestyle behaviors.

In measuring physical activity using IPAQ short form, possibly over reporting might have been occurred. This is known as a typical problem in several previous studies using the same questionnaire [77]. In this study, the cut-off point to achieve the physical activity recommendations was 600 MET-min/week, which is in accordance with the scorings guideline of the IPAQ questionnaire. However, in guidelines, different cut-off points or ranges were indicated [78–80]. Our cut-off point choice might have affected the outcome of the adherence to physical activity recommendations.

With regard to alcohol consumption, it could be that the results on alcohol are more a reflection of social drinkers and excessive drinkers, because some questions were focused on alcohol consumption, and non-drinkers might have found them to be not applicable to themselves. Although, similar questions were also applied to non-drinkers in prior research [81].

There was a probability that significant correlates could have occurred by chance due to multiple testing. However, by applying sequential multiple linear/logistic regression analyses, the chance on Type 1 error was rather small [58]. Moreover, given the adequate power, the *p*-values were highly significant which indicated that our estimates were relatively accurate.

## Conclusions

Overall, the participants of our study were more engaged in healthy lifestyle behaviors compared to other research, however, especially vegetable and fruit consumption were poor and should be considerably improved. The various lifestyle behaviors and the adherence to recommendations were influenced by different patterns of correlates, from which self-efficacy, attitudes, and intention were the strongest, although their contribution varied among the different lifestyle behaviors. Our findings emphasized that all examined lifestyle behaviors need to be encouraged in cancer survivors, with taken into consideration that each lifestyle behavior is influenced by a specific set of mainly motivational correlates.

## Abbreviations

ASE: Attitude-Social influence-Efficacy
BMI: body mass index
EORTC QLQ: European Organisation for Research and Treatment of Cancer
QoL: quality of life
HADS: Hospital Anxiety and Depression Scale
MAC: Mental Adjustment to Cancer scale
IPQ-R: Illness Perception Questionnaire Revised
SPSIR-R: S Short Social Problem Solving Inventory-Revised
IPAQ: International Physical Activity Questionnaire
MET: Metabolic Equivalent of Task

## References

[1] Blanchard CM, Courneya KS, Stein K. Cancer survivors’ adherence to lifestyle behavior recommendations and associations with health-related quality of life: Results from the American Cancer Society’s SCS-II. J Clin Oncol. 2008; doi:10.1200/JCO.2007.14.6217. 10.1200/JCO.2007.14.6217 18445845

[2] Davies NJ, Batehup L, Thomas R. The role of diet and physical activity in breast, colorectal, and prostate cancer survivorship: a review of the literature. Br J Cancer. 2011; doi 10.1038/bjc.2011.423. 10.1038/bjc.2011.423 PMC3251953 22048034

[3] Schlesinger S, Walter J, Hampe J, von Schonfels W, Hinz S, Kuchler T, Jacobs G, Schafmayer C, Nothlings U. Lifestyle factors and health-related quality of life in colorectal cancer survivors. Cancer Causes Control. 2014; doi: 10.1007/s10552-013-0313-y. 10.1007/s10552-013-0313-y 24158780

[4] Ford ES, Bergmann MM, Kroger J, Schienkiewitz A, Weikert C, Boeing H. Healthy living is the best revenge: findings from the European Prospective Investigation Into Cancer and Nutrition-Potsdam study. Arch Intern Med. 2009; doi: 10.1001/archinternmed.2009.237. 10.1001/archinternmed.2009.237 19667296

[5] Blanchard CM, Stein K, Baker F, Dent MF, Denniston MM, Courneya KS, Nehl E. Association between current lifestyle behaviors and health-related quality of life in breast, colorectal, and prostate cancer survivors. Psychol Health. 2004; doi: 10.1080/08870440310001606507.

[6] Baena Ruiz R, Salinas Hernandez P: Diet and cancer: Risk factors and epidemiological evidence. Maturitas. 2014; doi:10.1016/j.maturitas.2013.11.010. 10.1016/j.maturitas.2013.11.010 24374225

[7] Kushi LH, Kwan ML, Lee MM, Ambrosone CB (2007). Lifestyle factors and survival in women with breast cancer. J Nutr..

[8] Colditz GA, Wolin KY, Gehlert S. Applying what we know to accelerate cancer prevention. Sci Transl Med. 2012; doi: 10.1126/scitranslmed.3003218. 10.1126/scitranslmed.3003218 PMC3343638 22461645

[9] McLaughlin VH, Trentham-Dietz A, Hampton JM, Newcomb PA, Sprague BL. Lifestyle factors and the risk of a second breast cancer after ductal carcinoma in situ. Cancer Epidemiol Biomarkers Prev. 2014; doi:10.1158/1055-9965.EPI-13-0899. 10.1158/1055-9965.EPI-13-0899 PMC3951673 24403528

[10] Inoue-Choi M, Robien K, Lazovich D. Adherence to the WCRF/AICR guidelines for cancer prevention is associated with lower mortality among older female cancer survivors. Cancer Epidemiol Biomarkers Prev. 2013; doi: 10.1158/1055-9965.EPI-13-0054. 10.1158/1055-9965.EPI-13-0054 PMC3650116 23462914

[11] Schmid D, Leitzmann MF. Association between physical activity and mortality among breast cancer and colorectal cancer survivors: a systematic review and meta-analysis. Ann Oncol. 2014;doi: 10.1093/annonc/mdu012. 10.1093/annonc/mdu012 24644304

[12] Klosky JL, Tyc VL, Garces-Webb DM, Buscemi J, Klesges RC, Hudson MM. Emerging issues in smoking among adolescent and adult cancer survivors. A comprehensive review. Cancer. 2007; doi: 10.1002/cncr.23061. 10.1002/cncr.23061 17932906

[13] Wilson D, Parsons J, Wakefield M. The health-related quality-of-life of never smokers, ex-smokers, and light, moderate, and heavy smokers. Prev Med. 1999; doi: 10.1006/pmed.1999.0523. 10.1006/pmed.1999.0523 10479599

[14] Rock CL, Doyle C, Demark-Wahnefried W, Meyerhardt J, Courneya KS, Schwartz AL, Bandera EV, Hamilton KK, Grant B, McCullough M, et al. Nutrition and physical activity guidelines for cancer survivors. CA Cancer J Clin. 2012; doi: 10.3322/caac.21142. 10.3322/caac.21142 22539238

[15] Sehl M, Lu X, Silliman R, Ganz PA. Decline in physical functioning in first 2 years after breast cancer diagnosis predicts 10-year survival in older women. J Cancer Surviv. 2013; doi: 10.1007/s11764-012-0239-5. 10.1007/s11764-012-0239-5 PMC3568656 23232922

[16] LeMasters TJ, Madhavan SS, Sambamoorthi U, Kurian S. Health behaviors among breast, prostate, and colorectal cancer survivors: a US population-based case-control study, with comparisons by cancer type and gender. J Cancer Surviv. 2014; doi: 10.1007/s11764-014-0347-5. 10.1007/s11764-014-0347-5 PMC4892177 24532045

[17] Bellizzi KM, Rowland JH, Jeffery DD, McNeel T. Health behaviors of cancer survivors: examining opportunities for cancer control intervention. J Clin Oncol. 2005; doi: 10.1200/jco.2005.02.2343. 10.1200/JCO.2005.02.2343 16314649

[18] Williams K, Steptoe A, Wardle J. Is a cancer diagnosis a trigger for health behaviour change? Findings from a prospective, population-based study. Br J Cancer. 2013; doi: 10.1038/bjc.2013.254. 10.1038/bjc.2013.254 PMC3681023 23695026

[19] Coups EJ, Ostroff JS. A population-based estimate of the prevalence of behavioral risk factors among adult cancer survivors and noncancer controls. PrevMed. 2005; doi: 10.1016/j.ypmed.2004.09.011. 10.1016/j.ypmed.2004.09.011 15850868

[20] Eakin EG, Youlden DR, Baade PD, Lawler SP, Reeves MM, Heyworth JS, Fritschi L. Health behaviors of cancer survivors: data from an Australian population-based survey. Cancer Causes Control. 2007; doi: DOI10.1007/s10552-007-9033-5 18:881-894. 10.1007/s10552-007-9033-5 17638108

[21] Mayer DK, Terrin NC, Menon U, Kreps GL, McCance K, Parsons SK, Mooney KH. Health behaviors in cancer survivors. Oncol Nurs Forum. 2007; doi: 10.1188/07.ONF.643-651. 10.1188/07.ONF.643-651 17573323

[22] O’Neill SC, DeFrank JT, Vegella P, Richman AR, Henry LR, Carey LA, Brewer NT. Engaging in health behaviors to lower risk for breast cancer recurrence. PloS One. 2013; doi: 10.1371/journal.pone.0053607. 10.1371/journal.pone.0053607 PMC3543271 23326466

[23] Pronk NP, Anderson LH, Crain AL, Martinson BC, O’Connor PJ, Sherwood NE, Whitebird RR. Meeting recommendations for multiple healthy lifestyle factors. Prevalence, clustering, and predictors among adolescent, adult, and senior health plan members. Am J Prev Med. 2004; doi:10.1016/j.amepre.2004.04.022. 10.1016/j.amepre.2004.04.022 15275671

[24] De Vries H, Mudde AN (1998). Predicting stage transitions for smoking cessation applying the attitude-social influence-efficacy model. Psychol Health.

[25] De Vries H, Mudde AN, Dijkstra A, Willemsen MC. Differential beliefs, perceived social influences, and self-efficacy expectations among smokers in various motivational phases. Prev Med. 1998; doi: 10.1006/pmed.1998.0344. 10.1006/pmed.1998.0344 9808799

[26] De Vries H, Mudde AN, Leijs I, Charlton A, Vartiainen E, Buijs G (2003). The European smoking prevention framework approach (EFSA): An example of integral prevention. Health Educ Res..

[27] Fishbein M, Ajzen I (2010). Predicting And Changing Behavior. The Reasoned Action Approach.

[28] Charlier C, Van Hoof E, Pauwels E, Lechner L, Spittaels H, De Bourdeaudhuij I. The contribution of general and cancer-related variables in explaining physical activity in a breast cancer population 3 weeks to 6 months post-treatment. Psychooncology. 2013; doi: 10.1002/pon.2079. 10.1002/pon.2079 22052746

[29] Forbes CC, Blanchard CM, Mummery WK, Courneya KS. A comparison of physical activity correlates across breast, prostate and colorectal cancer survivors in Nova Scotia, Canada. Support Care Cancer. 2014;doi: 10.1007/s00520-013-2045-7. 10.1007/s00520-013-2045-7 24240648

[30] Husebo AM, Dyrstad SM, Soreide JA, Bru E. Predicting exercise adherence in cancer patients and survivors: a systematic review and meta-analysis of motivational and behavioural factors. J Clin Nurs. 2013; doi: 10.1111/j.1365-2702.2012.04322.x. 10.1111/j.1365-2702.2012.04322.x 23163239

[31] Kampshoff CS, Jansen F, van Mechelen W, May AM, Brug J, Chinapaw MJ, Buffart LM. Determinants of exercise adherence and maintenance among cancer survivors: a systematic review. Int J Behav Nutr Phys Act. 2014; doi: 10.1186/1479-5868-11-80. 10.1186/1479-5868-11-80 PMC4096543 24989069

[32] Madlensky L, Natarajan L, Flatt SW, Faerber S, Newman VA, Pierce JP. Timing of dietary change in response to a telephone counseling intervention: evidence from the WHEL study. Health Psychol. 2008; doi: 10.1037/0278-6133.27.5.539. 10.1037/0278-6133.27.5.539 PMC2562694 18823180

[33] Westmaas JL, Alcaraz KI, Berg CJ, Stein KD. Prevalence and Correlates of Smoking and Cessation-Related Behavior among Survivors of Ten Cancers: Findings from a Nationwide Survey Nine Years after Diagnosis. Cancer Epidemiol Biomarkers Prev. 2014; doi: 10.1158/1055-9965.EPI-14-0046. 10.1158/1055-9965.EPI-14-0046 25100826

[34] American Psychological Association. Ethical Principles of Psychologists and Code of Conduct. http://www.apa.org/ethics/code/index.aspx (2010). Accessed 10 dec 2015.

[35] Craig CL, Marshall AL, Sjostrom M, Bauman AE, Booth ML, Ainsworth BE, Pratt M, Ekelund U, Yngve A, Sallis JF, Oja P. International physical activity questionnaire: 12-country reliability and validity. Med Sci Sports Exerc. 2003; doi: 10.1249/01.MSS.0000078924.61453.FB. 10.1249/01.MSS.0000078924.61453.FB 12900694

[36] The International Physical Activity Questionnaire. https://sites.google.com/site/theipaq/. Accessed 8 march 2014.

[37] Vandelanotte C, De Bourdeaudhuij I, Philippaerts R, Sjöström M, Sallis J. Reliability and validity of a computerized and Dutch version of the International Physical Activity Questionnaire (IPAQ). J Phys Act Health. 2005; 2:63.

[38] Mudde AN, Willemsen MC, Kremers S, De Vries H (2006). Meetinstrumenten voor onderzoek naar roken en stoppen met roken.

[39] Loon AJM van, Veldhuizen H. Voortgangsrapportage 2003. Lokale en Nationale Monitor Volksgezondheid. http://www.rivm.nl/Documenten_en_publicaties/Wetenschappelijk/Rapporten/2004/augustus/Voortgangsrapportage_2003_Lokale_en_Nationale_Monitor_Volksgezondheid?sp=cml2bXE9ZmFsc2U7c2VhcmNoYmFzZT0zNDQ4MDtyaXZtcT1mYWxzZTs=&pagenr=3449. Accessed 8 march 2014.

[40] Brink CL van den, Ocke MC, Houben AW, Nierop P van, Droomers M. Validering van standaardvraagstelling voeding voor Lokale en Nationale Monitor Volksgezondheid. 2005. http://www.rivm.nl/Documenten_en_publicaties/Wetenschappelijk/Rapporten/2005/augustus/Validering_van_standaardvraagstelling_voeding_voor_Lokale_en_Nationale_Monitor_Volksgezondheid. Accessed 8 march 2014.

[41] Aaronson NK, Ahmedzai S, Bergman B, Bullinger M, Cull A, Duez NJ (1992). The European Organization for Research and Treatment of Cancer QLQ-C30: A quality-of-life instrument for use in international clinical trials in oncology. J Natl Cancer Inst.

[42] Fayers P, Bottomley A. Quality of life research within the EORTC—the EORTC QLQ-C30. Eur J Cancer. 2002; doi: 10.1016/s0959-8049(01)00448-8. 10.1016/s0959-8049(01)00448-8 11858978

[43] Fayers PM, Aaronson NK, Bjordal K, Groenvold M, Curran D, Bottomly A (2001). The EORTC QLQ-C30 Scoring Manual.

[44] Bjelland I, Dahl AA, Haug TT, Neckelmann D (2002). The validity of the Hospital Anxiety and Depression Scale. An updated literature review. J Psychosom Res.

[45] Zigmond AS, Snaith RP (1983). The hospital anxiety and depression scale. Acta Psychiatr Scand..

[46] Mitchell AJ, Meader N, Symonds P. Diagnostic validity of the Hospital Anxiety and Depression Scale (HADS) in cancer and palliative settings: a meta-analysis. J Affect Disord. 2010; doi 10.1016/j.jad.2010.01.067. 10.1016/j.jad.2010.01.067 20207007

[47] Braeken AP, Kempen GI, Watson M, Houben RM, Gils FC, Lechner L. Psychometric properties of the Dutch version of the Mental Adjustment to Cancer scale in Dutch cancer patients. Psychooncology. 2010; doi: 10.1002/pon.1628. 10.1002/pon.1628 19824025

[48] Watson M, Greer S, Young J, Inayat Q, Burgess C, Robertson B. Development of a questionnaire measure of adjustment to cancer: the MAC scale. Psychol Med. 1988; doi: 10.1007/s10549-005-9018-6. 10.1017/s0033291700002026 3363039

[49] Watson M, Homewood J. Mental Adjustment to Cancer Scale: psychometric properties in a large cancer cohort. Psychooncology. 2008; doi: 10.1002/pon.1345. 10.1002/pon.1345 18626853

[50] Broadbent E, Petrie KJ, Main J, Weinman J. The brief illness perception questionnaire. J Psychosom Res. 2006; doi: 10.1016/j.jpsychores.2005.10.020. 10.1016/j.jpsychores.2005.10.020 16731240

[51] Weinman J, Petrie KJ, Moss-Morris R, Horne R. The illness perception questionnaire: A new method for assessing the cognitive representation of illness. Psychol Health. 1996; doi: 10.1080/08870449608400270.

[52] D’Zurilla TJ, Nezu AM, Maydeu-Olivares A. Social Problem-Solving Inventory – Revised. 2007. http://www.mhs.com/product.aspx?gr=cli&prod=spsi-r&id=overview#top. Accessed 8 march 2014.

[53] Smeets T, Kremers SP, Brug J, de Vries H. Effects of tailored feedback on multiple health behaviors. Ann Behav Med. 2007; doi: 10.1093/her/cyl10. 10.1007/BF02879892 17447863

[54] Smit ES, de Vries H, Hoving C. Determinants of practice nurses’ intention to implement a new smoking cessation intervention: the importance of attitude and innovation characteristics. J Adv Nurs. 2013; doi: 10.1111/jan.12153. 10.1111/jan.12153 23600904

[55] Bakker EC, Nijkamp MD, Sloot C, Berndt NC, Bolman CA. Intention to abstain from smoking among cardiac rehabilitation patients: the role of attitude, self-efficacy, and craving. J Cardiovasc Nurs. 2015; doi: 10.1097/JCN.0000000000000156. 10.1097/JCN.0000000000000156 24831728

[56] Berndt N, Bolman C, Froelicher ES, Mudde A, Candel M, de Vries H, Lechner L. Effectiveness of a telephone delivered and a face-to-face delivered counseling intervention for smoking cessation in patients with coronary heart disease: a 6-month follow-up. J Behav Med. 2014; doi: 10.1007/s10865-013-9522-9. 10.1007/s10865-013-9522-9 23760610

[57] Ronda G, Van Assema P, Brug J (2001). Stages of change, psychological factors and awareness of physical activity levels in The Netherlands. Health Promot Int..

[58] Tabachnick BG, Fidell LS (2001). Using Multivariate Statistics.

[59] Stevinson C, Lydon A, Amir Z. Adherence to physical activity guidelines among cancer support group participants. Eur J Cancer Care. 2014; doi: 10.1111/ecc.12145. 10.1111/ecc.12145 24127843

[60] Bertheussen GF, Oldervoll L, Kaasa S, Sandmael JA, Helbostad JL. Measurement of physical activity in cancer survivors-a comparison of the HUNT 1 Physical Activity Questionnaire (HUNT 1 PA-Q) with the International Physical Activity Questionnaire (IPAQ) and aerobic capacity. Support Care Cancer. 2013; doi: 10.1007/s00520-012-1530-8. 10.1007/s00520-012-1530-8 22797861

[61] Cuevas BT, Hughes DC, Parma DL, Trevino-Whitaker RA, Ghosh S, Li R, Ramirez AG. Motivation, exercise, and stress in breast cancer survivors. Support Care Cancer. 2014; doi: 10.1007/s00520-013-2038-6. 10.1007/s00520-013-2038-6 PMC3943705 24249424

[62] Del Valle MO, Martin-Payo R, Lana A, Garcia JB, Folgueras MV, Lopez ML. Behavioural cancer risk factors in women diagnosed with primary breast cancer. J Adv Nurs. 2014; doi: 10.1111/jan.12433. 10.1111/jan.12433 24773512

[63] Weaver KE, Foraker RE, Alfano CM, Rowland JH, Arora NK, Bellizzi KM, Hamilton AS, Oakley-Girvan I, Keel G, Aziz NM. Cardiovascular risk factors among long-term survivors of breast, prostate, colorectal, and gynecologic cancers: a gap in survivorship care? J Cancer Surviv. 2013; doi: 10.1007/s11764-013-0267-9. 10.1007/s11764-013-0267-9 PMC3756807 23417882

[64] Berg CJ, Thomas AN, Mertens AC, Schauer GL, Pinsker EA, Ahluwalia JS, Khuri FR. Correlates of continued smoking versus cessation among survivors of smoking-related cancers. Psychooncology. 2012; doi: 10.1002/pon.3077. 10.1002/pon.3077 PMC3425712 22488864

[65] Grimmett C, Bridgewater J, Steptoe A, Wardle J: Lifestyle and quality of life in colorectal cancer survivors. Qual Life Res. 2011; doi: 10.1007/s11136-011-9855-1. 10.1007/s11136-011-9855-1 21286822

[66] Bidstrup PE, Dalton SO, Christensen J, Tjonneland A, Larsen SB, Karlsen R, Brewster A, Bondy M, Johansen C. Changes in body mass index and alcohol and tobacco consumption among breast cancer survivors and cancer-free women: a prospective study in the Danish Diet, Cancer and Health Cohort. Acta Oncol. 2013; doi: 10.3109/0284186X.2012.746466. 10.3109/0284186X.2012.746466 23244678

[67] Dumalaon-Canaria JA, Hutchinson AD, Prichard I, Wilson C. What causes breast cancer? A systematic review of causal attributions among breast cancer survivors and how these compare to expert-endorsed risk factors. Cancer Causes Control. 2014; doi: 10.1007/s10552-014-0377-3. 10.1007/s10552-014-0377-3 24771106

[68] Kwan ML, Kushi LH, Weltzien E, Tam EK, Castillo A, Sweeney C, Caan BJ. Alcohol consumption and breast cancer recurrence and survival among women with early-stage breast cancer: the life after cancer epidemiology study. J Clin Oncol. 2010; doi: 10.1200/JCO.2010.29.2730. 10.1200/JCO.2010.29.2730 PMC2988633 20805458

[69] Chan AM, von Muhlen D, Kritz-Silverstein D, Barrett-Connor E. Regular alcohol consumption is associated with increasing quality of life and mood in older men and women: the Rancho Bernardo Study. Maturitas. 2009; doi: 10.1016/j.maturitas.2009.01.005. 10.1016/j.maturitas.2009.01.005 PMC2681249 19232847

[70] Lang I, Wallace RB, Huppert FA, Melzer D. Moderate alcohol consumption in older adults is associated with better cognition and well-being than abstinence. Age Ageing. 2007; doi: 10.1093/ageing/afm001. 10.1093/ageing/afm001 17353234

[71] Milliron BJ, Vitolins MZ, Tooze JA. Usual Dietary Intake among Female Breast Cancer Survivors Is Not Significantly Different from Women with No Cancer History: Results of the National Health and Nutrition Examination Survey, 2003-2006. J Acad Nutr Diet. 2013; doi: 10.1016/j.jand.2013.08.015. 10.1016/j.jand.2013.08.015 PMC4000572 24169415

[72] Ocke MC, Buurma-Rethans EJM, Boer de EJ, Wilson-van den Hooven C, Etemad-Ghameshlou Z, Drijvers JJMM, Rossum van CTM. Diet of community-dwelling ouder adults. Dutch National Food Consumption Survey Older adults 2010-2012. http://www.rivm.nl/Documenten_en_publicaties/Wetenschappelijk/Rapporten/2013/oktober/Diet_of_community_dwelling_older_adults_Dutch_National_Food_Consumption_Survey_Older_adults_2010_2012. Accessed 18 jan 2014.

[73] Dijkstra SC, Neter JE, Brouwer IA, Huisman M, Visser M. Adherence to dietary guidelines for fruit, vegetables and fish among older Dutch adults; the role of education, income and job prestige. J Nutr Health Aging. 2014; doi: 10.1007/s12603-013-0402-3. 10.1007/s12603-013-0402-3 PMC12878990 24522461

[74] Spring B, King AC, Pagoto SL, Van Horn L, Fisher JD. Fostering multiple healthy lifestyle behaviors for primary prevention of cancer. Am Psychol. 2015; doi: 10.1037/a0038806. 10.1037/a0038806 PMC4626078 25730716

[75] Bradbury KE, Appleby PN, Key TJ. Fruit, vegetable, and fiber intake in relation to cancer risk: findings from the European Prospective Investigation into Cancer and Nutrition (EPIC). Am J Clin Nutr. 2014; doi: 10.3945/ajcn.113.071357. 10.3945/ajcn.113.071357 24920034

[76] Low CA, Beckjord E, Bovbjerg DH, Dew MA, Posluszny DM, Schmidt JE, Lowery AE, Nutt SA, Arvey SR, Rechis R. Correlates of positive health behaviors in cancer survivors: Results from the 2010 LIVESTRONG Survey. J Psychosoc Oncol. 2014; doi: 10.1080/07347332.2014.955243. 10.1080/07347332.2014.955243 PMC5772769 25176347

[77] Lee PH, Macfarlane DJ, Lam TH, Stewart SM. Validity of the International Physical Activity Questionnaire Short Form (IPAQ-SF): a systematic review. Int J Behav Nutr Phys Act. 2011; doi: 10.1186/1479-5868-8-115. 10.1186/1479-5868-8-115 PMC3214824 22018588

[78] Haskell WL, Lee IM, Pate RR, Powell KE, Blair SN, Franklin BA, Macera CA, Heath GW, Thompson PD, Bauman A. Physical activity and public health: updated recommendation for adults from the American College of Sports Medicine and the American Heart Association. Circulation. 2007; doi: 10.1161/CIRCULATIONAHA.107.185649. 10.1161/CIRCULATIONAHA.107.185649 17671237

[79] Nelson ME, Rejeski WJ, Blair SN, Duncan PW, Judge JO, King AC, Macera CA, Castaneda-Sceppa C. Physical activity and public health in older adults: recommendation from the American College of Sports Medicine and the American Heart Association. Circulation. 2007; doi:10.1161/CIRCULATIONAHA.107.185650. 10.1161/CIRCULATIONAHA.107.185650 17671236

[80] Garber CE, Blissmer B, Deschenes MR, Franklin BA, Lamonte MJ, Lee IM, Nieman DC, Swain DP. American College of Sports Medicine position stand. Quantity and quality of exercise for developing and maintaining cardiorespiratory, musculoskeletal, and neuromotor fitness in apparently healthy adults: guidance for prescribing exercise. Med Sci Sports Exerc. 2011; doi: 10.1249/MSS.0b013e318213fefb. 10.1249/MSS.0b013e318213fefb 21694556

[81] Schulz DN, Kremers SP, Vandelanotte C, van Adrichem MJ, Schneider F, Candel MJ, de Vries H. Effects of a web-based tailored multiple-lifestyle intervention for adults: a two-year randomized controlled trial comparing sequential and simultaneous delivery modes. J Med Internet Res 2014; doi: 10.2196/jmir.3094. 10.2196/jmir.3094 PMC3936298 24472854

